# Positive Effects of Enhanced Recovery After Surgery (ERAS) Combined with a Dual-Attending Surgeon (DAS) Approach in Patients Operated on for Adolescent Idiopathic Scoliosis (AIS)

**DOI:** 10.3390/jcm14207334

**Published:** 2025-10-17

**Authors:** Sara Knutsson, Vojtech Capek, Helena Brisby, Olof Westin

**Affiliations:** 1 Department of Orthopedics, Institute of Clinical Sciences, Sahlgrenska Academy, University of Gothenburg, SE413 45 Gothenburg, Sweden; vojtech.capek@vgregion.se (V.C.); helena.brisby@vgregion.se (H.B.); olof.westin@gmail.com (O.W.); 2 Spine Surgery Unit, Orthopedic Clinic, Sahlgrenska University Hospital, SE413 45 Gothenburg, Sweden

**Keywords:** adolescent idiopathic scoliosis, posterior spinal fusion, enhanced recovery after surgery, dual-attending surgeons, perioperative outcomes

## Abstract

**Background/Objectives**: Enhanced recovery after surgery (ERAS) is intended to facilitate recovery after major surgery and was implemented at our institution together with a dual-attending surgeon (DAS) approach for adolescent idiopathic scoliosis (AIS) surgery. The aim of the present study was to investigate whether this change in care influenced hospital length of stay (LOS), operative time, and opioid consumption compared to the preceding approach. **Methods**: This is a retrospective cohort study. A post-intervention (ERAS/DAS) group of 50 AIS patients undergoing posterior spinal fusion (PSF) surgery obtained a multimodal analgesic regimen, including intrathecal morphine, and were operated on by dual-attending surgeons. This group was compared with 50 patients in a pre-intervention (pre-ERAS/DAS) group. **Results**: Compared to the pre-intervention group, ERAS/DAS patients had a reduced LOS, with a mean difference of −1.5 days (95% CI [−2.0; −1.0]; *p* < 0.0001). Operative time was shorter, with a mean difference of −48 min (95% CI [−62; −33]; *p* < 0.0001). There was a reduction in the in-hospital total opioid consumption, with a mean difference of −328 mg (95% CI [−406; −250]; *p* < 0.0001). Additionally, the length of postoperative intensive care unit (ICU) stay was reduced, with a mean difference of −11.5 h (95% CI [−12.7; −10.3]; *p* < 0.0001). **Conclusions**: The ERAS/DAS concept implemented at our institution resulted in reductions in hospital LOS, operative time, postoperative ICU stay, and in-hospital opioid consumption. Decreasing operative time and length of stay allows more patients to receive access to crucial treatment and enables more efficient use of finite resources.

## 1. Introduction

Paediatric scoliosis surgery is a complex and demanding procedure that carries a substantial risk for complications. According to the Scoliosis Research Society (SRS) Morbidity and Mortality Committee report, which includes 19,360 patients, the overall complication rate in paediatric scoliosis surgery is 10.2% [1]. Although the highest complication rates are found in the neuromuscular and congenital scoliosis groups (17.9% and 10.3%, respectively), the complication rate for adolescent idiopathic scoliosis (AIS) surgery is still significant at 6.3%. Multiple studies have shown that risk factors for complications include, amongst others, long operating time, excessive blood loss, and allogenic blood transfusion [2,3,4,5]. Minimising complications is in the interest of all spine surgeons.

The concept of enhanced recovery after surgery (ERAS) was introduced by the ERAS Study Group in 2001 [6]. It is a multimodal, multidisciplinary perioperative care pathway facilitating recovery for patients undergoing major surgery. Although sometimes associated with fast-track surgery, the original aim of the ERAS Study Group was to focus on the quality, rather than the speed, of recovery. While it has been implemented in a variety of procedures, the underlying rationale behind ERAS is the same: to reduce the stress response as well as surgery-related pain and to maintain homeostasis following surgery. ERAS has been extensively studied and has reduced hospital length of stay (LOS) in several medical fields, e.g., general surgery, urology, and gynaecology [7].

During the last decade, the ERAS concept has gained increasing attention in the field of orthopaedic and spine surgery. A recent meta-analysis and systematic review of ERAS in AIS included 10 studies and almost 2000 patients. The use of ERAS-type protocols was found to significantly reduce the length of stay without increasing complication or readmission rates [8]. Another trend in spinal surgery is the concept of dual-attending surgeons (DAS), which has been associated with a reduction in operative time, blood loss, and perioperative recovery [9,10,11]. A 2024 meta-analysis of DAS in spinal deformity surgery showed that it reduces complications, operative time, and LOS, but not blood loss [12].

To enhance the quality of care, and also with the expressed intention to increase the number of surgeries performed, an ERAS protocol was introduced at our institution in 2017. Together with the ERAS protocol, a dual-attending surgeon approach (ERAS/DAS) was put into practice. The aim of the present study was to investigate whether this change in care influenced hospital LOS, operative time, and/or opioid consumption compared to the preceding (pre-ERAS/DAS) approach.

## 2. Materials and Methods

### 2.1. Study Design

This is a retrospective study at a tertiary spine surgery centre. The reporting adheres to the STROBE statement guidelines for observational studies. The study was approved by the Swedish Ethical Review Authority (2024-06352-1). The power analysis determined that a minimum of 100 patients was required to detect a difference in the primary outcome between the groups with 80% power.

An ERAS/DAS protocol was introduced at our institution in January 2018 and fully implemented by May 2018. After May 2018, all AIS patients aged 10–25 years old undergoing primary PSF surgery were operated on according to the ERAS/DAS protocol. Beyond ERAS/DAS, there were no systematic changes regarding surgical technique, anaesthetic practice, or postoperative care during 2016–2020. The pre-ERAS/DAS group consisted of 50 consecutive patients undergoing surgery just before the introduction of ERAS/DAS, between January 2016 and December 2017. The ERAS/DAS group consisted of 50 consecutive patients undergoing surgery after ERAS/DAS was fully implemented, from May 2018 to March 2020. Study participants were identified through a query to the surgical planning system. All patients with AIS between 10 and 25 years old who had undergone primary PSF surgery and followed the routine protocol at the time of surgery were included in the study.

### 2.2. Enhanced Recovery After Surgery (ERAS)

The ERAS protocol was designed in collaboration with the anaesthesiology department and was based on a protocol used by the scoliosis spine surgeons at Rikshospitalet in Oslo, Norway. All the professions involved in the perioperative care of these patients were engaged in developing the ERAS protocol. Beyond perioperative pharmacological changes, the protocol included instructions for the pre- and postoperative phases, focusing on early mobilisation and normalisation of bowel movements. The discharge criteria were not changed.

Prior to surgery, patients > 12 years and weighing > 40 kg were given 90 mg etoricoxib and 600 mg gabapentin, whereas patients < 12 years or <40 kg were only given gabapentin 5 mg/kg. After induction of anaesthesia, intravenous (IV) betamethasone at 0.3 mg/kg (max 16 g) and a bolus of IV tranexamic acid at 30 mg/kg were administered; tranexamic acid infusion at 10 mg/kg/h was then continued until the end of the surgery.

Before the skin incision, intrathecal morphine 5 µg/kg (max 250 µg) was administered by one of the surgeons, usually at the L3–4 interlaminar space. Before emergence from anaesthesia, IV paracetamol 15 mg/kg (max 1 g) and iv fentanyl 1 µg/kg were administered. For patients who did not receive NSAIDs preoperatively, IV ketorolac 0.5 mg/kg was also given. Upon arrival at the postoperative ICU, patients received extended- and short-release oxycodone, as well as paracetamol and gabapentin if needed.

According to the ERAS protocol, patients were expected to leave the postoperative ICU within 6 h and were encouraged to stand up prior to discharge to the ward. Patients were monitored for 12 h for respiratory complications after intrathecal morphine administration. Physiotherapy was started on postoperative day 1 and included rehabilitation milestones for each day. Postoperative pain management at the ward consisted of paracetamol, extended- and short-release oxycodone and/or codeine. All opioids were converted to equivalent doses of oxycodone.

### 2.3. Dual-Attending Surgeons (DAS)

All patients in the ERAS/DAS group were operated on by dual-attending surgeons with several years of experience in scoliosis surgery.

### 2.4. Pre-ERAS/DAS Group

Prior to the implementation of the ERAS protocol, the standard procedure of PSF surgery for AIS included information given orally about the surgical procedure, pain management strategies, and rehabilitation. Preoperatively, the patients were given extended-release oxycodone and paracetamol. The surgery was performed by a single attending surgeon, with a junior spine surgeon or resident as assistant. Patients were usually monitored at the postoperative intensive care unit (ICU) until the day after surgery. Mobilisation was started upon return to the ward.

Postoperative pain management consisted of paracetamol and patient-controlled analgesia (PCA) for IV administration of oxycodone. After PCA was discontinued, the patient received extended- and short-release oxycodone and/or codeine. The total dose of IV oxycodone given through PCA was converted to the equivalent dose of oral oxycodone (1:1.5 IV oxycodone:PO oxycodone).

### 2.5. Outcome Measure

Patients’ demographics and curve characteristics were retrieved from medical records and radiographs, respectively. The screw density is a ratio of the number of pedicle screws used in the spinal fusion divided by twice the number of fused vertebrae. The primary outcome was hospital LOS (days). Secondary outcomes were operative time (min, from administration of intrathecal morphine to wound closure), estimated blood loss (mL, from visual estimation), allogenic blood transfusion rate (%), length of postoperative ICU stay (hours), in-hospital opioid consumption (mg), and 30-day readmission rate (%). All outcome measurements were collected retrospectively from medical records and the surgical planning system.

### 2.6. Statistical Analysis

Continuous variables were reported as means and standard deviations (SD). The dataset did not contain any extreme outliers. The *t*-test was used for between-groups comparisons. The 95% confidence intervals were calculated and bootstrapped if a normal distribution was not assumed. Categorical variables were presented as counts and proportions (%), and a Chi-square test was used for between-groups comparisons. All statistical tests were two-sided, and alpha was set to the 0.05 level. The statistical analyses were performed using SPSS v 29.0.2.0 (IBM, Chicago, IL, USA).

## 3. Results

### 3.1. Baseline and Intraoperative Characteristics

The age, gender, BMI, Cobb angle, type of fusion, as well as levels fused, and screw density were similar between groups (Table 1). The average age in the ERAS/DAS group was 16.0 years, and 38 patients (76%) were female. The average age of pre-ERAS/DAS patients was 15.2 years, and 40 patients (80%) were female. The mean thoracic and lumbar Cobb angles in the ERAS/DAS group were 46 and 39 degrees, respectively, whereas in the pre-ERAS/DAS group, they were 48 and 39 degrees. In the ERAS/DAS group, 25 patients (50%) underwent selective thoracic fusion, 14 (28%) selective lumbar fusion, and 11 (22%) double-curve fusion. The corresponding numbers for the pre-ERAS/DAS group were selective thoracic fusion *n* = 30 (60%), selective lumbar fusion *n* = 9 (18%), and double-curve fusion *n* = 11 (22%). The screw density was 78% for both groups.

### 3.2. Primary and Secondary Outcomes

There was a reduction in hospital LOS in the ERAS/DAS compared to the pre-ERAS/DAS group, with a mean difference of −1.5 days (95% CI [−2.0; −1.0]; *p* < 0.0001; Table 2). Operative time was shorter in the ERAS/DAS compared to the pre-ERAS/DAS group, with a mean difference of −48 min (95% CI [−62; −33], *p* < 0.0001). Also, the length of postoperative ICU stay was reduced with a mean difference of −11.5 h (95% CI [−12.7; −10.3]; *p* < 0.0001). Additionally, there was a reduction in the in-hospital total opioid (oxycodone) consumption with a mean difference of −328 mg (95% CI [−406; −250]; *p* < 0.0001). The average daily opioid consumption was reduced with a mean difference of −49 mg (95% CI [−60; −37]; *p* < 0.0001). The effect sizes for the findings were generally high.

The total estimated blood loss did not differ between groups. The mean difference in the ERAS/DAS compared to the pre-ERAS/DAS group was −36 mL (95% CI [−121; 49]; *p* = 0.20). The rate of blood transfusion did not differ either. In both groups, four patients (8%) received allogenic blood transfusion.

Finally, there was no statistically significant difference in the 30-day readmission rate. One (2%) ERAS/DAS patient and two (4%) pre-ERAS/DAS patients were readmitted within 30 days after the surgery (*p* = 1.00). The ERAS/DAS patient was readmitted one day post-discharge because of nausea and vomiting, which resolved spontaneously. The pre-ERAS/DAS patients were readmitted because of early postoperative infection. Both infections resolved after a single revision surgery and antibiotic treatment for 6–12 weeks.

## 4. Discussion

The main finding of the present study is that the implementation of the ERAS protocol and dual-attending surgeon approach resulted in a significant reduction in the operative time, a shorter postoperative ICU stay, and earlier discharge compared to the pre-ERAS/DAS approach. Also, the in-hospital opioid consumption was lower in the ERAS/DAS group. To our knowledge, the present study is the first to combine these two approaches and compare a pre- to a post-intervention cohort. The present study demonstrates that the combination of these two concepts improves the results of AIS surgery.

The results from the present study are in line with findings from previous studies implementing either an ERAS-type concept or a dual-attending surgeon approach [13,14,15,16]. In their 2021 meta-analysis and systematic review, Gadiya et al. report LOS data from seven different studies, ranging from 2.17 to 3.7 days, with a mean difference of −1.44 days [8]. Thus, the decrease in LOS is comparable with the findings of the present study, with a reported LOS reduction of −1.5 days. The complexity of healthcare logistics makes it difficult to fully compare LOS between countries, or even between hospitals in the same country. Therefore, we believe that a reduction in LOS is more important than LOS itself.

Halanski et al. reviewed 106 AIS patients, of which 24 were operated on by dual-attending surgeons. With dual-attending surgeons, operative time was shorter, the drop in haemoglobin levels was smaller, and patients’ length of stay was shorter [10]. Chan and Kwan prospectively studied the perioperative outcome of PSF for AIS in 60 patients. The DAS group had significantly reduced operative time, blood loss, and allogenic blood transfusion [11]. Sarwahi et al. reviewed 519 cases of AIS patients undergoing PSF. They found that standard-volume surgeons had better outcomes with a DAS approach, but that the positive effect for high-volume surgeons was minimal [17].

At our institution, before the implementation of the ERAS protocol and the dual-attending surgeon approach, PSF for AIS was considered a full-day project in the operating room (OR). The operative time had already decreased compared to a couple of years ago, from usually around 4 h down to 3 h, but the preoperative preparation in the OR still lasted almost as long. Furthermore, patients stayed at the postoperative ICU until the next day. To achieve optimisation and standardisation of the workflow around these patients, cooperation between staff members and coordination of tasks were required. The goal was to perform the needed preoperative procedures simultaneously and not one after another. As this succeeded, we were able to double the number of PSF surgeries for AIS performed in one day. However, the generalizability of these results at any given institution depends on factors such as resources, OR logistics, and patient populations.

Patients in the ERAS/DAS group had significantly lower opioid consumption than their pre-ERAS/DAS counterparts. We believe that this decrease was largely due to the changes made to the perioperative pain management protocols. Intrathecal morphine combined with gabapentin has previously been found to result in a reduced consumption of oral opioids after PSF for AIS [18]. Also, NSAIDs have been shown to reduce opioid consumption in paediatric surgery in general [19]. Munro et al. found, when studying the effect of low-dose ketorolac following PSF, that patients who received ketorolac had lower pain scores and lower opioid consumption compared to controls [20]. In a literature review of multimodal pain management in paediatric spine surgery, Shah et al. concluded that, although each pain medication has its own specific benefits, the best results occur when the medications are used in combination [21].

Limitations of the present study include the retrospective design. The study investigated patients operated on during a time span of 4 years (2016–2020). To our knowledge, there were no major changes during this period apart from the alterations due to the implementation of the ERAS protocol and dual-attending surgeons. Nevertheless, it is possible that there are unaccounted-for factors affecting the analysed parameters.

A major limitation of the study is the lack of pain scores and/or patient-reported outcome measures (PROM) data, which would have added another dimension to the study. As the study is based on existing information in medical records, we unfortunately have no means of collecting PROM data in retrospect. In hindsight, pain scores and/or PROM data should have been collected for these patients when implementing the ERAS/DAS protocol. Although there were no changes regarding opioids given as needed, the patients had lower opioid consumption and returned home sooner in the ERAS/DAS group. Our interpretation of this was a decrease in postoperative pain levels. However, whether the patients experienced less pain or not remains to be proven.

## 5. Conclusions

The ERAS concept, together with a dual-attending surgeon approach implemented at our institution, resulted in reductions in hospital LOS, operative time, postoperative ICU stay, and in-hospital opioid consumption. Decreasing operative time and length of stay allows for more patients to obtain access to crucial treatment and enables more efficient use of finite resources.

## Figures and Tables

**Table 1:** Patients’ demographics.

	ERAS/DAS *n* = 50	Pre-ERAS/DAS *n* = 50	*p*
Gender			
Female (%)	38 (76)	40 (80)	
Male (%)	12 (24)	10 (20)	0.81
Age, yrs	16.0 (3.2)	15.2 (2.9)	0.20
Weight, kg	57.0 (10.8)	56.7 (9.2)	0.88
Length, cm	167.5 (10.4)	164.6 (8.0)	0.12
BMI	20.1 (2.8)	20.8 (2.7)	0.19
Cobb angle			
Thoracic	46 (15)	48 (16)	0.62
Lumbar	39 (12)	39 (13)	0.96
No. fused vertebras	8.6 (2.3)	8.8 (1.9)	0.54
Type of fusion (%)			
STF	25 (50)	30 (60)	
SLF	14 (28)	9 (18)	
DCF	11 (22)	11 (22)	0.46
Screw density, %	78 (12)	78 (10)	0.93

Mean (SD), *n* (%). STF = selective thoracic fusion, SLF = selective lumbar fusion, DCF = double-curve fusion. Independent samples *t*-test and *X*^2^ were used for comparison of continuous and categorical variables, respectively. *p* value < 0.05 was statistically significant.

**Table 2:** Outcomes comparison between ERAS/DAS and pre-ERAS/DAS group.

	ERAS/DAS *n* = 50	Pre-ERAS/DAS *n* = 50	Mean Difference (95% CI)	Effect Size ^1^	*p*
Operative time, min	130 (31)	178 (40)	−48 (−62; −33)	−1.32 (−1.75; −0.89)	<0.0001
Estimated blood loss, mL	332 (175)	368 (249)	−36 (−121;49)	−0.17 (−0.56; 0.23)	0.20
Blood transfusion	4 (8)	4 (8)	0		1.00
ICU stay, hours	6.5 (2.0)	18.0 (3.7)	−11.5 (−12.7; −10.3)	−3.82 (−4.48; −3.16)	<0.0001
Length of stay, days	4.2 (1.3)	5.7 (1.3)	−1.5 (−2.0; −1.0)	−1.17 (−1.60; −0.75)	<0.0001
30-day readmission	1 (2)	2 (4)	1		1.00
Average opioid consumption per day ^2^, mg	28 (13)	77 (39)	−49 (−60; −37)	−1.66 (−2.11; −1.20)	<0.0001
Total in-hospital opioid consumption ^2^, mg	122 (78)	450 (265)	−328 (−406; −250)	−1.68 (−2.13; −1.22)	<0.0001

Mean (SD), *n* (%). ^1^ Cohen’s d (SD). ^2^ milligrams oxycodone equivalent. Independent samples *t*-test and *X*^2^ were used for comparison of continuous and categorical variables, respectively. *p* value < 0.05 was statistically significant.

## Data Availability

The datasets presented in this study are available from the corresponding author upon reasonable request.

## Abbreviations

The following abbreviations are used in this manuscript:

ERAS	Enhanced recovery after surgery
DAS	Dual-attending surgeons
AIS	Adolescent idiopathic scoliosis
LOS	Length of stay
PSF	Posterior spinal fusion
ICU	Intensive care unit
SRS	Scoliosis Research Society
PCA	Patient-controlled analgesia
SD	Standard deviation
STF	Selective thoracic fusion
SLF	Selective lumbar fusion
DCF	Double-curve fusion
OR	Operating room
